# Free will and paranormal beliefs

**DOI:** 10.3389/fpsyg.2014.00281

**Published:** 2014-04-02

**Authors:** Ken Mogi

**Affiliations:** Sony Computer Science LaboratoriesTokyo, Japan

**Keywords:** free will, paranormal beliefs, consciousness, gender, illusion

## Abstract

Free will is one of the fundamental aspects of human cognition. In the context of cognitive neuroscience, various experiments on time perception, sensorimotor coordination, and agency suggest the possibility that it is a robust illusion (a feeling independent of actual causal relationship with actions) constructed by neural mechanisms. Humans are known to suffer from various cognitive biases and failures, and the sense of free will might be one of them. Here I report a positive correlation between the belief in free will and paranormal beliefs (UFO, reincarnation, astrology, and psi). Web questionnaires involving 2076 subjects (978 males, 1087 females, and 11 other genders) were conducted, which revealed significant positive correlations between belief in free will (theory and practice) and paranormal beliefs. There was no significant correlation between belief in free will and knowledge in paranormal phenomena. Paranormal belief scores for females were significantly higher than those for males, with corresponding significant (albeit weaker) difference in belief in free will. These results are consistent with the view that free will is an illusion which shares common cognitive elements with paranormal beliefs.

## Introduction

The ability to choose one's own action is one of the most salient features of our daily experience. In the literature, the status and nature of free will is still debated (Haggard, 2011; Smith, 2011). Causal determinism is the fundamental assumption in the physical sciences, and provides an implicit framework for chemical and biological sciences. The libertarian position on free will (Clarke, 2003) denies causal determinism, at least in humans and other agents possessing free will. Taking the determinist position in its strict sense, on the other hand, could lead to the negation of free will. When considering the findings of modern and contemporary sciences, the compatibility with causal determinism becomes a fundamental constraint on the nature of free will. The concept of free will, even when its existence is maintained, must somehow be made compatible with causal determinism.

The exact nature of the compatibility of free will with causal determinism is an open question (Van Inwagen, 1975; Kane, 1998, 2011). The compatibilist position on free will does not necessarily assume that both causal determinism and free will are true. However, the compatibilist position is commonly held to imply the truth of some form of determinism and the possession of free will (Fischer, 2009).

One of the possible ways to make free will compatible with causal determinism is to assume that it is an “illusion,” which implies that “the experience of consciously willing an action is not a direct indication that the conscious thought has caused the action” (Wegner, 2003). The notion that free will is an illusion dissociated from actual causation is not new. Hume (1739) defined human “will” as “nothing but the internal impression we feel and are conscious of, when we knowingly give rise to any new motion of our body, or new perception of our mind.” Here, I take the position that free will is an illusion dissociated from actual causation, although the relevance of data reported below are not necessarily limited to this particular position.

Evidence suggests that the feeling that one has free will arises as a result of coordinated activities of neural circuits in the brain. The mere presence of action is not sufficient for a sense of free will. When the motor cortex was stimulated directly during an operation, the produced movements were not accompanied by a sense of free will causing the action (Penfield, 1975). The possession of agency is an important element of free will. In the alien hand syndrome (Goldstein, 1908; Banks et al., 1989), the subject loses the sense of agency for the limb and feels that it has a “will of its own.” In split-brain patients, the left brain (which is responsible for verbal report) is unaware of the cause of actions induced by communications to the right hemisphere (Gazzaniga, 1995), resulting in the impairment of the reportable sense of free will.

The disruption of free will is a pathological condition affecting the subject's overall wellbeing. Patients with Schizophrenia suffer from avolition, in which the patient experiences low drive and lack of motivation to pursue goals (Andreasen, 1982; Messinger et al., 2011). In the dissociative identity disorder (Reinders et al., 2003; American Psychiatric Association, 2013), multiple personalities alternatingly take control, with compartmentalized knowledge and memories, resulting in a loss of an integrated sense of free will.

The disruption of a normal sense of free will can sometimes involve more than one agent. It is known that the brain's bookkeeping mechanism associated with agency sometimes misplaces cause and effect, especially in a situation involving interactions between individuals. For example, the facilitated communication is a controversial method in which the facilitator supports the hand/arm of an impaired person, helping the individual to communicate (Jacobson et al., 1995). It has been shown that the intentional origin of action can be misplaced, with the agency of action attributed to a wrong person (Wegner and Wheatley, 1999; Wegner et al., 2003). Facilitated communication is not an isolated case. The belief in external agents can lead to a belief in bizarre dissociations of perceived authorship in such cases as trance channeling and spirit possession (Wegner, 2002).

The integration of information regarding sensorimotor contingency is an important element in the construction of the sense of free will, in which time is an essential parameter. In normal subjects, the conscious will to produce an action is perceived to precede the actual initiation of action, while brain activities leading to the action physically occurs before the conscious realization of the urge to move (Libet, 1985). Libet's results have been interpreted to indicate that the decision to initiate action takes place unconsciously before the conscious perception of it occurs, suggesting that conscious processing is an epiphenomenon or at best a vetoing mechanism.

Alternative interpretations of Libet's scheme have been proposed. Schurger et al. (2012) liken the neural decision of spontaneous action to “tipping over the first in a row of dominoes,” setting into motion “a cascade that is ballistic, but not deterministic.” In this scheme, the premotor activities are characterized by ongoing fluctuations, which contributes to the buildup for action initiation through a leaky stochastic accumulator. The gradual 1-s to 2-s increase in neural activities preceding spontaneous movements are common to both vertebrates (Kornhuber and Deecke, 1965; Romo and Schultz, 1987) and invertebrates (Kagaya and Takahata, 2010), suggesting the universality of such a process. Miller and Schwarz (2014), citing the experiment of Matsuhashi and Hallet (2008), argue for a graded model of conscious decision making, as an alternative to Libet's all-or-none model.

Summarizing the works reviewed thus far, the evidence is compatible with the idea that free will is an illusion, with the disruption of the related processes resulting in anomalies and pathologies.

In a previous study, it was found that there was a significant correlation between the subject's sense of free will and paranormal worldviews (Mogi, 2013). In contrast, there was no significant correlation between paranormal beliefs and the subject's views on qualia, another essential element of phenomenal experience. Thus, although free will and qualia are two salient features of conscious experience, their correlations with paranormal beliefs are significantly different. Some authors argue that qualia are illusions (e.g., Dennett, 1993). The difference in correlations with paranormal beliefs suggests that qualia and free will are cognitively different, even if both of them are indeed illusions. Studying the correlations of the sense of free will with paranormal beliefs in more detail would facilitate the clarification of the cognitive factors involved.

Paranormal phenomena are defined as those that, if genuine, would violate basic limiting principles of science (Broad, 1953). It is difficult to maintain paranormal beliefs in a manner consistent with the findings of contemporary sciences. However, paranormal beliefs (e.g., UFOs, reincarnation, psychic powers, and astrology) persist in society in spite of the best evidence against them. Paranormal beliefs among the general population are persistent according to the Gallup poll. In 1976, 17, 24, and 9% of people living in the USA believed in astrology, UFOs, and reincarnation, respectively, whereas in 1997, the ratios of believers were 37, 30, and 25%, respectively (cited in French and Wilson, 2007). The 2005 Gallup poll (http://www.gallup.com/poll/16915/three-four-americans-believe-paranormal.aspx) found that 25% of living in the USA believed in astrology, 20 % believed in reincarnation, while 41% believed in ESP (extrasensory perception). Thus, beliefs in paranormal phenomena continue to be an ingredient of folk psychology.

Some studies have suggested that there are gender differences in paranormal beliefs. Specifically, female subjects show stronger belief in paranormal phenomena (e.g., ESP and astrology) compared to male subjects (Irwin, 1993; Rice, 2003). On the other hand, previous studies on belief in free will (Viney et al., 1982; Rakos et al., 2008) did not specifically investigate the gender differences.

Misinterpreting or ignoring empirical evidence or acting on wrong assumptions (e.g., choosing one's action based on astrology readings) can be maladaptive, since such strategies do not have empirical or causal basis. It is thus an interesting question why and how paranormal beliefs persist. Are paranormal beliefs the result of ignorance? Do they represent the subject's cognitive biases? Cognitive biases can be adaptive in some contexts (Gigerenzer and Goldstein, 1996). Given the rate of prevalence among the general public, is it possible that paranormal beliefs (and/or the cognitive tendencies correlating with them) are adaptive in some contexts?

Some studies have revealed brain processes correlated with paranormal beliefs. Citing evidence for the effects of stimulation and hallucinations caused by vascular anomalies, activities in the temporal lobe have been suggested to correlate with religious and mystical experiences (Persinger, 1983). An EEG (electroencephalogram) study revealed that believers in paranormal phenomena showed relatively higher right hemispheric activation and reduced hemispheric asymmetry of functional complexity (Pizzagalli et al., 2000). Disruptions of multisensory information integration from one's own body at the temporo-parietal junction (TPJ) have been suggested to correlate with out-of-body experience, an important element of some paranormal phenomena (Blanke and Arzy, 2005).

Few studies have specifically looked at belief in free will and paranormal beliefs in a common context, as they have been considered separate. It is thus interesting to study how these elements of cognition are related, in view of the possible correlations between the two.

Here I investigate the correlations between paranormal beliefs and the sense of free will. Two aspects of belief in free will were put to question. The first is the subject's belief in the existence of free will as a theory. The second is the subject's belief in possessing free will in the practical executions of action in their daily lives. Four elements of paranormal beliefs, i.e., reincarnation, astrology, UFO, and psi were investigated. These elements are based on the generally accepted paranormal belief scale (Tobacyk and Milford, 1983; Tobacyk, 2004), in which seven-point questionnaires were adopted as an appropriate method of investigation. In addition, the subjects' knowledge of paranormal phenomena was investigated, to differentiate the correlations of paranormal beliefs and paranormal knowledge with the belief in free will.

## Methods

The web-based questionnaire was conducted in Japanese. Participation was accepted for 6 days, from 17:00 3rd December 2013, JST (Japan Standard Time) to 17:00 8th December 2013, JST. The author's twitter account (@kenichiromogi, with 522256 and 523956 followers at the beginning and end of web survey, respectively) was used to recruit subjects for participation. The advertising tweet introduced and called for participation in a “survey on free will,” with the URL of the questionnaire. There was no mention of paranormal beliefs at this stage. There were nominally 2113 entries at the end of the survey period. When checked for irregularities, 33 subjects were found to have made entries twice, while two subjects made three entries. The multiple entries were found to be consistent within a particular subject, and were treated as single entry per subject for further analysis. Seven subjects entered their age with Chinese characters (a common irregularity found in the Japanese webspace). These entries were corrected into equivalent Arabic numerals. After filtering and rectifying these irregularities, data for 2076 subjects (978 males, 1087 females, and 11 other genders, with age average of 37.5 and standard deviation of 11.3) were submitted to further analysis.

The subjects were first asked two free will related questions. Question 1: Do you agree with the statement “humans have free will?” Question 2: Do you agree with the statement “in everyday life, you are actually choosing your actions freely?” The subjects answered questions 1 and 2 in 7-point scales (1: Not at all so, 2: Not so, 3: Slightly not so, 4: Neutral, 5: Slightly so, 6:So, 7: Very much so). Question 1 and 2 were designed to assess the subjects' “theoretical” and “practical” beliefs in the existence of free will, respectively. The belief in free will (theory) question corresponded to “the Free Will and Determinism-General Will Questions” in Rakos et al. (2008), which is comprised of 14 items (e.g., “Free will is a basic part of human nature”), while the belief in free will (practice) questions corresponded to the “Free Will and Determinism-Personal Will Questions” in Rakos et al. (2008), which is comprised of eight items (e.g., “I am in charge of the decisions I make”).

After the free will questions, the subjects answered questions regarding their belief in paranormal worldviews. In the questionnaire, no explanation was given as to the relevance of paranormal questions to the question of free will. There were no contexts suggesting any correlations between the belief in free will and paranormal beliefs. The comments after the completion of the questionnaire suggested that the subjects did not in general suspect the nature of the relevance of paranormal belief questions in a survey on free will. Indeed, some of the subjects expressed their perplexity at being asked the paranormal questions.

There were four paranormal belief questions. Question 3: Do you agree with the statement “reincarnation exists?” Question 4: Do you agree with the statement “the divinations of astrology are significant?” Question 5: Do you agree with the statement “UFOs are flying to the earth, with aliens on them?” Question 6: Do you agree with the statement “psychic powers such as precognition, clairvoyance, and psychokinesis exist?” The subject answered these questions in 7-point scales (1: Not at all so, 2: Not so, 3: Slightly not so, 4: Neutral, 5: Slightly so, 6: So, 7: Very much so). The four themes covered in the paranormal belief questions are based on the generally accepted paranormal belief scale (Tobacyk and Milford, 1983; Tobacyk, 2004), with the 7-point scale questioning methodology.

The paranormal belief questions were followed by the corresponding paranormal knowledge questions 7, 8, 9, 10 (Table 1), in which the subjects answered the number of items that they knew out of 10 presented. The knowledge items asked in these questions were chosen to have global significance, while being relevant to the Japanese populace. The subjects completed the questionnaire by answering their age, gender (male, female, or others) and indicating their informed consent in the understanding that the entries would be statistically processed, to be used in a scientific investigation without revealing the identities of the participants.

**Table 1:** **Question items for four categories of paranormal beliefs**.

**Category**	**(1)**	**(2)**	**(3)**	**(4)**	**(5)**	**(6)**	**(7)**	**(8)**	**(9)**	**(10)**
Reincarnation (Question 7)	Book of the dead	Village of reincarnation	“Out on a limb”	Bardo	Past life regression therapy	Regression hypnosis	Reincarnated lama	Karma	Brain Weiss	Ian Stevenson
Astrology (Question 8)	“The wise man is stronger than the stars” ^*^1	Horoscope	Zodiac	Astrological signs	Cardinal sign	Ecliptic	Ruling planet	Ophiuchus	Jonathan Cainer	Jean Petit
UFO (Question 9)	Captured alien photo	Area 51	Greys	Roswell incident	Majestic 12	Cattle mutilation	Alien abduction	Crop circle	George Adamski	Joseph Allen Hynek
Psi (Question 10)	Psychokinesis	ESP	Psychometry	Pyrokinesis	Thoughtography	Poltergeist	Rapping	Zenar cards	Uri Geller	Derren Brown

* 1 Phrase attributed to Thomas Aquinas.

The subjects answered the number of items they had a sufficient knowledge to explain to others out of the 10 items listed in the table. The knowledge items asked in these questions were chosen to have global significance, while being relevant to the Japanese populace.

## Methods: ethics statement

The experimental protocol was submitted to and approved by the brain and cognitive sciences ethics committee of Sony Computer Science Laboratories.

## Results

Two-tailed student's *t*-tests were conducted to test significances. The average theoretical and practical belief scores in free will were 5.25 and 4.38, respectively, with standard deviations of 1.56 and 1.57, respectively. The nominal score was significantly higher for the theoretical belief compared to the practical belief (*t* = 17.9, *p* = 6.9 × 10^−64^). There was a significant correlation between the theoretical and practical beliefs in free will (*r* = 0.529, *t* = 24.1, *p* = 2.90 × 10^−113^). The average belief scores were 3.47, 3.19, 3.94, and 4.26 for reincarnation, astrology, UFO, and psi respectively, with standard deviations of 2.07, 1.83, 1.85, and 1.81, respectively. The sum of the four belief scores (henceforth referred to as the “paranormal belief score”) was calculated for each subject and used for further analysis. The average of paranormal belief score was 14.6, with a standard deviation of 5.9. The average paranormal knowledge scores were 0.99, 0.95, 1.79, and 2.65 for reincarnation, astrology, UFO, and psi respectively, with standard deviations of 1.68, 1.81, 2.31, and 2.54, respectively. The sum of the four knowledge scores (henceforth referred to as the “paranormal knowledge score”) was calculated for each subject and used for further analysis. The average of paranormal knowledge score was 6.28, with a standard deviation of 6.68. There was a significant correlation between paranormal belief scores and paranormal knowledge scores (*r* = 0.223, *t* = 10.4, *p* = 9.93 × 10^−25^).

**Figure 1:**
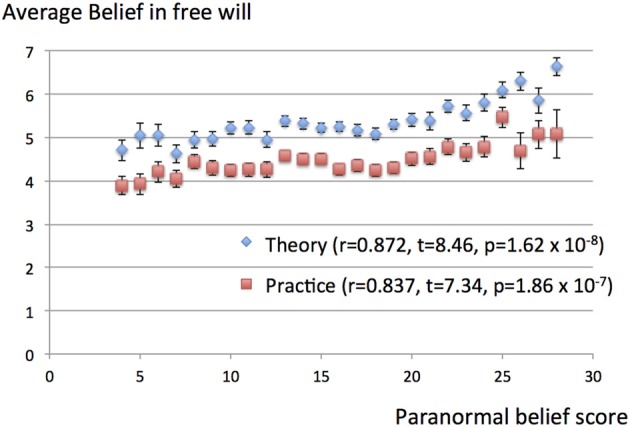
**Average belief in free will for particular paranormal belief scores.** The scores for belief in free will (theory and practice) are average and plotted for subjects with particular paranormal belief scores. The possible range of scores for belief in free will was 1–7, while that for paranormal belief was 4–28. Vertical bars represent standard errors of the mean. The correlation coefficients between the paranormal belief score and beliefs in free will (theory and practice) were 0.872 (*t* = 8.46, *p* = 1.62 × 10^−8^) and 0.837 (*t* = 7.34, *p* = 1.86 × 10^−7^), respectively.

**Figure 2:**
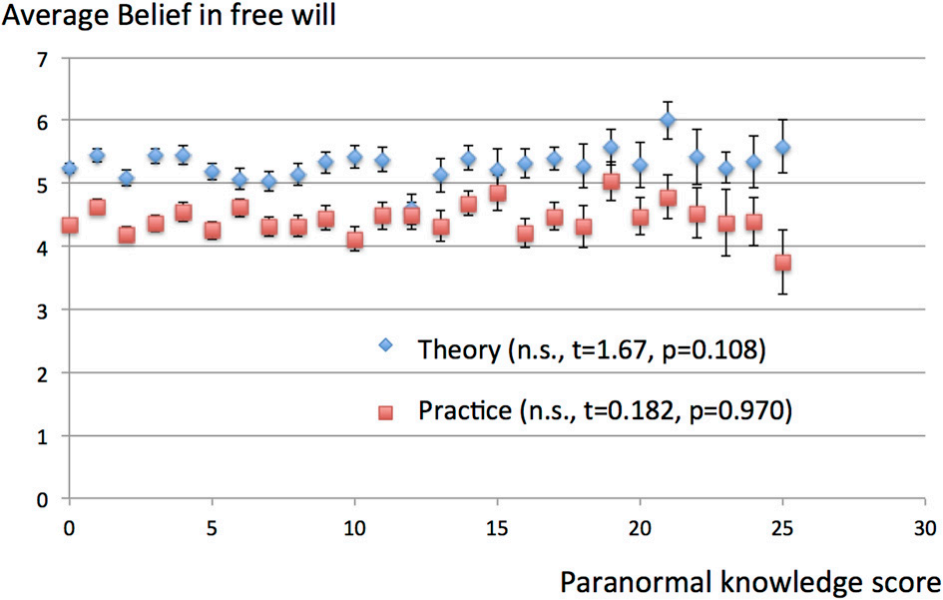
**Average belief in free will for paranormal knowledge scores.** The scores for belief in free will (theory and practice) are averaged and plotted for subjects with particular paranormal knowledge scores. The possible range of scores for belief in free will was 1–7, while that for paranormal knowledge was 0–40. Vertical bars represent standard errors of the mean. There were no significant correlations between the paranormal knowledge score and beliefs in free will [theory (*t* = 1.67, *p* = 0.108) and practice (*t* = 0.182, *p* = 0.970)].

There were significant correlations between the paranormal belief scores and the theoretical and practical beliefs in free will. Taking the data of each subject as individual samples, the correlation coefficient between the theoretical belief in free will and paranormal belief score was 0.164 (*t* = 7.57, *p* = 5.58 × 10^−14^), while that between the practical belief in free will and paranormal belief score was 0.123 (*t* = 5.64, *p* = 1.93 × 10^−8^). Taking the data for each subject as individual samples, there were no significant correlations between paranormal knowledge scores and the theoretical (*t* = 0.346, *p* = 0.729) or practical (*t* = 0.697, *p* = 0.486) beliefs in free will. These results are summarized in Table 2. The graph plotting the average free will belief scores for subjects with particular paranormal belief scores reveal a monotonously increasing relationship (Figure 1), with correlation coefficients between the paranormal belief scores and beliefs in free will of 0.872 (*t* = 8.46, *p* = 1.62 × 10^−8^) and 0.837 (*t* = 7.34, *p* = 1.86 × 10^−7^) for theoretical and practical beliefs, respectively. The graph plotting the average free will belief scores for subjects with particular paranormal knowledge scores reveal a flat relationship (Figure 2). There were no significant correlations between paranormal knowledge scores and the theoretical (*t* = 1.67, *p* = 0.108) or practical (*t* = 0.182, *p* = 0.970) beliefs in free will.

**Table 2:** **Correlations between belief in free will (theory/practive) and paranormal belief/knowledge scores**.

	**Paranormal belief score**	**Paranormal knowledge score**
Belief in free will (theory)	*r* = 0.164 (*t* = 7.57, *p* = 5.58 × 10^−14^)	n.s. (*t* = 0.346, *p* = 0.729)
Belief in free will (practice)	*r* = 0.123 (*t* = 5.64, *p* = 1.93 × 10^−8^)	n.s. (*t* = 0.697, *p* = 0.486)

There were significant correlations of theoretical and practical belief in free will with paranormal belief scores, with r = 0.164 (t = 7.57, p = 5.58 × 10^−14^) and r = 0.123 (t = 5.64, p = 1.93 × 10^−8^), respectively. There were no significant correlations of theoretical and practical belief in free with paranormal knowledge scores, with t = 1.67, p = 0.108 and t = 0.182, p = 0.970, respectively.

**Figure 3:**
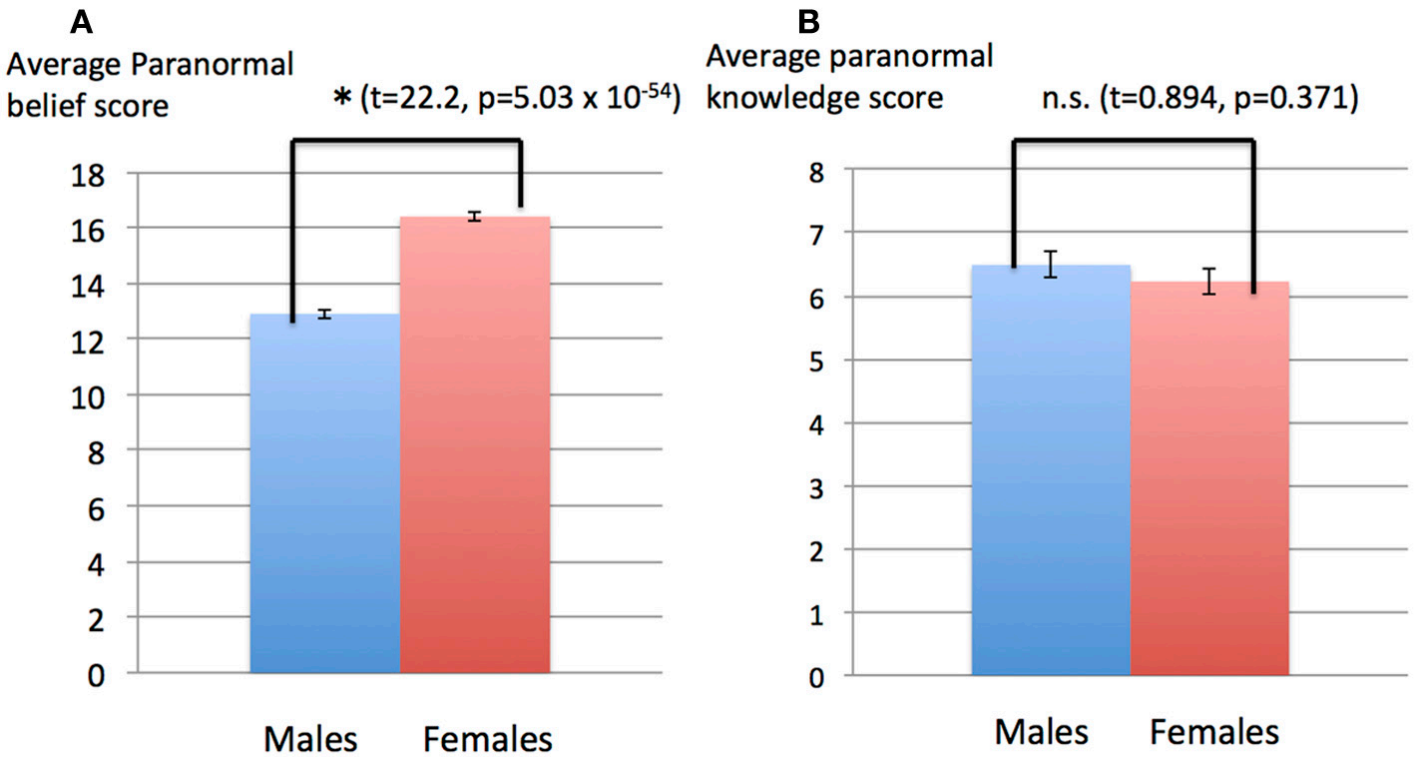
**Gender difference in paranormal belief and knowledge scores. (A)** Paranormal belief scores for male and female subjects. The average paranormal belief scores for males and females were 12.9 and 16.6, respectively, with standard deviations of 5.2 and 5.4, respectively. Vertical bars represent standard errors of the mean. The difference of paranormal belief scores between the male and female subjects was significant (*t* = 22.2, *p* = 5.03 × 10^−54^). **(B)** Paranormal knowledge scores for male and female subjects. The average paranormal knowledge scores for males and females were 6.49 and 6.23, respectively, with standard deviations of 6.62 and 6.66, respectively. Vertical bars represent standard errors of the mean. The difference of paranormal knowledge scores between the male and female subjects was not significant (*t* = 0.894, *p* = 0.371).

**Figure 4:**
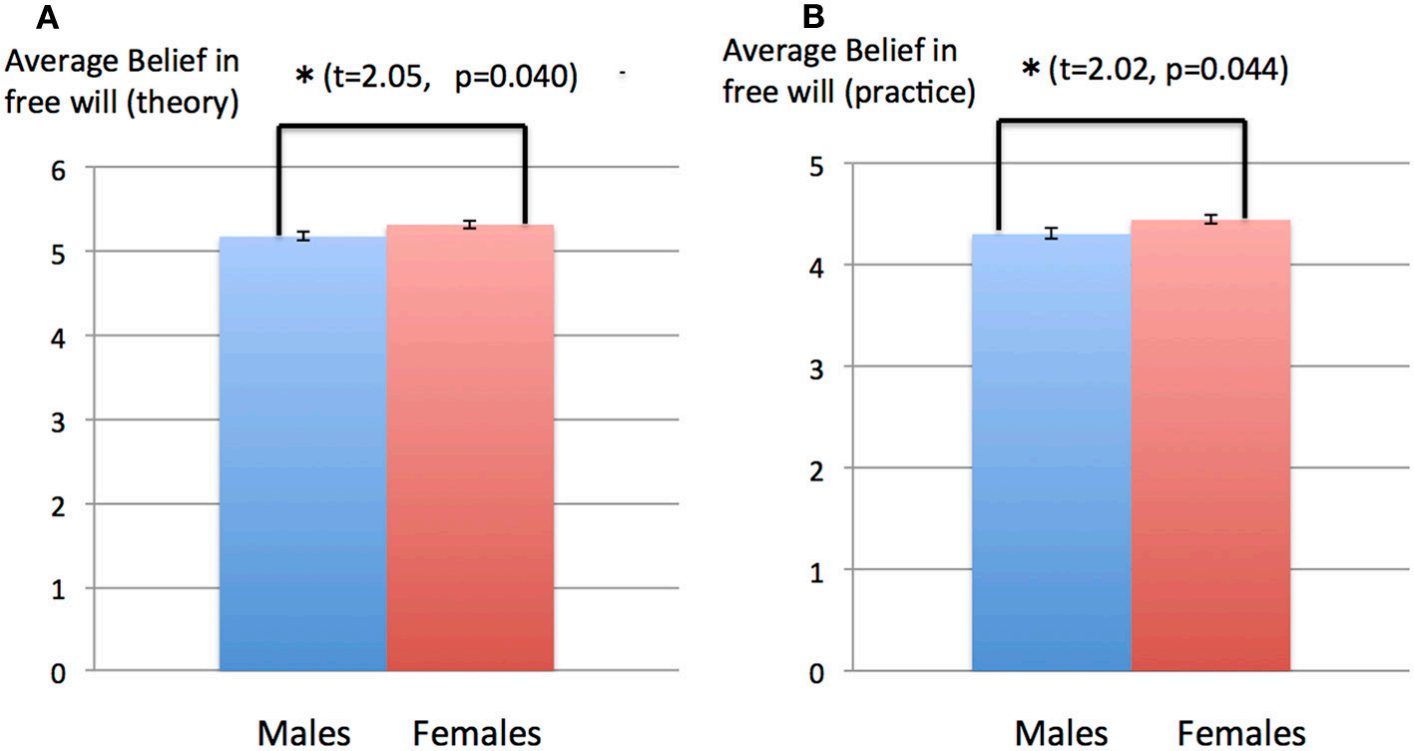
**Gender difference in the belief in free will (theory and practice). (A)** Belief in free will (theory) for male and female subjects. The average belief in free will (theory) were 5.18 and 5.32 for male and female subjects, respectively, with standard deviations of 1.68 and 1.44, respectively. Vertical bars represent standard errors of the mean. The difference between males and females was significant (*t* = 2.05, *p* = 0.040). **(B)** Belief in free will (practice) for male and female subjects. The average belief in free will (practice) were 4.31 and 4.45, respectively, with standard deviations of 1.62 and 1.52. Vertical bars represent standard errors of the mean. The difference between males and females was significant (*t* = 2.02, *p* = 0.044).

Gender comparison was conducted for males and females only, as the number of “other genders” entries were too small to arrive at statistically significant results (11 other genders in comparison with 978 males and 1087 females). Analysis of data revealed some significant differences between male and female subjects. The average paranormal belief scores for males and females were 12.9 and 16.6, respectively, with standard deviations of 5.2 and 5.4, respectively (Figure 3A). The difference of paranormal belief scores between the male and female subjects was significant (*t* = 22.2, *p* = 5.03 × 10^−54^). The average paranormal knowledge scores for males and females were 6.49 and 6.23, respectively, with standard deviations of 6.62 and 6.66, respectively (Figure 3B). The difference of paranormal knowledge scores between male and female subjects was not significant (*t* = 0.894, *p* = 0.371). The average beliefs in free will (theory) were 5.18 and 5.32 for male and female subjects, respectively, with standard deviations of 1.68 and 1.44, respectively (Figure 4A). The difference between males and females was significant (*t* = 2.05, *p* = 0.040). The average belief scores in free will (practice) for males and females were 4.31 and 4.45, respectively, with standard deviations of 1.62 and 1.52 (Figure 4B). The difference between males and females was significant (*t* = 2.02, *p* = 0.044).

## Discussion

This study has addressed the relation between the subjects' perception of free will and paranormal beliefs. The method of present study can only reveal correlations between cognitive tendencies in a large number of subjects. The number of items asked in this study as regards free will (theory and practice) was small compared to the more extensive surveys on free will (e.g., Rakos et al., 2008). These limitations should be taken into account when interpreting the data reported here.

The method of recruiting subjects (through tweets of the author) might have introduced some bias in the participant base. By the nature of tweets of the authors account, the follower base is likely to reflect a general interest in themes of neuroscience and cognition. The followers, however, would not be necessarily actively interested in paranormal views, as the author's account rarely touches on that particular theme. When calling for participation in a survey on free will, the tweets did not mention questions on paranormal beliefs. Thus, the significant correlations between belief in free will and paranormal beliefs reported here are not likely to be the result of the recruited subjects specifically interested in paranormal phenomena.

The average belief score for free will in theory was nominally significantly higher than that for in practice. However, the two measures exhibited similar tendencies in relation to paranormal belief and knowledge scores. There was a significant correlation between the subjects' assessment of free will and paranormal belief scores. On the other hand, the correlation between belief in free will and paranormal knowledge scores was not significant.

It is possible that the significant correlations between the subjects' assessment of free will and paranormal belief scores are the result of some subjects having a general bias to give high “belief” scores in the Lickert scale, but not high “knowledge” scores. If such is the case, it is possible that the significant correlations found here are not specifically between belief in free will and paranormal belief scores. Analysis based on the present set of data cannot exclude this possibility. On the other hand, the results of Mogi (2013), which found no significant correlations between the subjects' evaluations of qualia and paranormal belief while finding significant correlations between free will and paranormal belief scores, are consistent with the interpretation that there is indeed a specific correlation between free will and paranormal belief.

Although there were significant correlations between belief in free will (theory or practice) and paranormal belief scores, the range of beliefs in free will was not necessarily large. The differences between the highest and lowest average belief scores for subjects with particular paranormal belief scores (Figure 1) were 1.92 and 1.57 in the Likert scale for the theoretical and practical beliefs in free will, respectively. The practical significances (if any) of the positive correlation between belief in free will and paranormal belief scores should be considered in view of these actual changes involved.

There were significant gender differences between male and female subjects, with female subjects tending to have more paranormal belief. This finding is consistent with previous research (Irwin, 1993; Rice, 2003). Female subjects reported more belief in free will compared to male subjects, albeit by a smaller margin compared to that for paranormal beliefs.

In view of the positive correlation between belief in free will and paranormal beliefs, it is interesting to consider the possible adaptive or maladaptive values for subjects holding these beliefs.

Holding a paranormal belief seems to indicate a lack of critical thinking, as evidence from modern and contemporary sciences are generally against the existence of paranormal phenomena. Believers in the paranormal were found to perform less well on probability estimation tasks (Blackmore and Trościanko, 1985). In an experiment of psychokinesis, the subjects had the tendency to believe that their trials were successful, even when they were statistically not different from chance (Benassi et al., 1979). People often fall victim to the illusion of control, in which subjects often confuse skill and luck (Langer, 1975). Henslin (1967) found that when playing dice, people tended to throw softly when they wanted low numbers, while throwing hard for high numbers. When subjects had the opportunity to bet before and after the dices were thrown (but before the outcome was known), the subjects placed larger bets when betting before rather than after the toss (Strickland et al., 1966). Illusions of control are likely to occur in settings characterized by personal involvement, familiarity, foreknowledge of desired outcomes, and a focus on success (Thompson, 1999). The illusion of free will also has social impacts. The Dunning-Krueger effect describes a situation where subjects tend to have overly favorable views of their abilities in social and intellectual domains (Kruger and Dunning, 1999).

Believers in reincarnation sometimes claim that the good or bad nature of deeds in one's lifetime is reflected in the next life after reincarnation. The “just world” hypothesis assumes that actions and outcomes must have the same valence (Lerner, 1980), where the subjects expect, for example, that noble deeds are rewarded accordingly, or that victims are partly to blame for what they suffer, even when there are no such correlations from objective points of view. The prevalence of such cognitive biases might correlate with the persistence of paranormal beliefs (e.g., reincarnation).

If holding paranormal belief or belief in free will is correlated with these cognitive biases and failures, why do they persist? If free will and paranormal worldview are positively correlated, as this study suggests, are there common themes to be considered as regards the adaptive values of these illusions?

Jahoda (1958) identified five criteria of positive mental health: Positive attitudes toward the self; the ability to grow, develop, and self-actualize; autonomy; environmental mastery in work and social relationships; and integration. Positive illusions have been suggested to promote mental health in the context of Jahoha (Taylor and Brown, 1988, 1994). Having positive illusions has been tied to reports of happiness, with perception of self-esteem, self-confidence, and sense of control (Freedman, 1978). Having illusions in free will or paranormal phenomena might have been adaptive in these contexts, even when they do not necessarily reflect the reality accurately.

It has been suggested that as humans evolved, those who believed in internal agency might have been more effective in controlling their environment (Rakos, 2004). Keynes (1936) discussed the “animal spirits” behind human behavior, arguing that humans decided to do something as the result of a spontaneous urge to action rather than inaction. Having a sense of free will would suggest that the subject is proactive, or at least not shy of taking actions. Having paranormal worldviews might result in the subject taking actions (e.g., based on astrological readings) even when they are based on misconceptions. Illusions such as free will and paranormal beliefs might have been adaptive in that they made the subjects more proactive.

### Conflict of interest statement

The author declares that the research was conducted in the absence of any commercial or financial relationships that could be construed as a potential conflict of interest.

## References

[B1] American Psychiatric Association. (2013). Diagnostic and Statistical Manual of Mental Disorders, 5th Edn. Arlington, VA: American Psychiatric Publishing

[B2] Andreasen N. C. (1982). Negative symptoms in schizophrenia: definition and reliability. Arch. Gen. Psychiatry 39, 784–788 10.1001/archpsyc.1982.04290070020005 7165477

[B3] Banks G. Short P. Martínez A. J. Latchaw R. Ratcliff G. Boller F. (1989). The alien hand syndrome: clinical and postmortem findings. Arch. Neurol. 46, 456–459 10.1001/archneur.1989.00520400116030 2705906

[B4] Benassi V. A. Sweeney P. D. Drevno G. E. (1979). Mind over matter: perceived success at psychokinesis. J. Pers. Soc. Psychol. 37, 1377–1386 10.1037/0022-3514.37.8.1377

[B5] Blackmore S. Trościanko T. (1985). Belief in the paranormal: Probability judgements, illusory control, and the ‘chance baseline shift’. Br. J. Psychol. 76, 459–468 10.1111/j.2044-8295.1985.tb01969.x

[B6] Blanke O. Arzy S. (2005). The out-of-body experience: disturbed self-processing at the temporo-parietal junction. Neuroscientist 1, 16–24 10.1177/1073858404270885 15632275

[B7] Broad C. D. (1953). The relevance of psychical research to philosophy, in Philosophy and Parapsychology, ed Ludwig J. (Buffalo, NY: Prometheus), 43–63

[B8] Clarke R. (2003). Libertarian Accounts of Free Will. Oxford: Oxford University Press 10.1093/019515987X.001.0001

[B9] Dennett D. C. (1993). Consciousness Explained. London: Penguin

[B10] Fischer J. M. (2009). Four Views on Free Will (Great Debates in Philosophy). Oxford: Wiley-Blackwell

[B11] Freedman J. L. (1978). Happy People: What Happiness is, Who has it, and Why. New York, NY: Harcourt Brace Jovanovich

[B12] French C. C. Wilson K. (2007). Cognitive factors underlying paranormal beliefs and experiences, in Tall tales about the Mind and Brain. Separating Fact from Fiction, ed Della Sala S. (Oxford: Oxford University Press), 3–22 ISBN: 978-0-19-856877-3

[B13] Gazzaniga M. S. (ed.). (1995). Consciousness and the cerebral hemispheres, in The Cognitive Neurosciences (Cambridge: MIT Press), 1391–1400

[B56] Gigerenzer G. Goldstein D. G. (1996). Reasoning the fast and frugal way: models of bounded rationality. Psychol. Rev. 103, 650–669 10.1037/0033-295X.103.4.650 8888650

[B14] Goldstein K. (1908). Zur Lehre von der motorischen Apraxie. J. Psychol. Neurol. (Lpz.), XI, 169–187, 270–283.

[B15] Haggard P. (2011). Decision time for free will. Neuron 69, 404–406 10.1016/j.neuron.2011.01.028 21315252

[B16] Henslin J. M. (1967). Craps and magic. Am. J. Sociol. 73, 316–330 10.1086/224479

[B17] Hume D. (1739). A Treatise of Human Nature. London: John Noon

[B18] Irwin H. J. (1993). Belief in the paranormal: a review of empirical literature. J. Soc. Psychical Res. 87, 1–39

[B19] Jacobson J. W. Mulick J. A. Schwartz A. A. (1995). A history of facilitated communication: Science, pseudoscience, and antiscience science working group on facilitated communication. Am. Psychol. 50, 750 10.1037/0003-066X.50.9.750

[B20] Jahoda M. (1958). Current Concepts of Positive Mental Health. New York, NY: Basic Books 10.1037/11258-000

[B21] Kagaya K. Takahata M. (2010). Readiness discharge for spontaneous initiation of walking in crayfish. J. Neurosci. 30, 1348–1362 10.1523/JNEUROSCI.4885-09.2010 20107061 PMC6633775

[B22] Kane R. (1998). The Significance of Free Will. New York, NY: Oxford University Press

[B23] Kane R. (ed.). (2011). The Oxford Handbook of Free Will. New York, NY: Oxford University Press 10.1093/oxfordhb/9780195399691.001.0001

[B24] Keynes J. M. (1936). The General Theory of Employment, Interest and Money. London: Macmillan

[B25] Kornhuber H. H. Deecke L. (1965). Changes in brain potentials with willful and passive movements in humans: the readiness potential and reafferent potentials. Pflugers Arch. 284, 1–17 10.1007/BF00412364 14341490

[B26] Kruger J. Dunning D. (1999). Unskilled and unaware of it: how difficulties in recognizing one's own incompetence lead to inflated self-assessments. J. Pers. Soc. Psychol. 77, 1121 10.1037/0022-3514.77.6.1121 10626367

[B27] Langer E. J. (1975). The illusion of control. J. Pers. Soc. Psychol. 32, 311 10.1037/0022-3514.32.2.31116634679

[B28] Lerner M. J. (1980). The Belief in a Just World: A Fundamental Delusion. New York, NY: Plenum Press 10.1007/978-1-4899-0448-5

[B29] Libet B. (1985). Unconscious cerebral initiative and the role of conscious will in voluntary action. Behav. Brain Sci. 8, 529–566 10.1017/S0140525X00044903

[B30] Matsuhashi M. Hallet M. (2008). The timing of the conscious intention to move. Eur. J. Neurosci. 28, 2344–2351 10.1111/j.1460-9568.2008.06525.x 19046374 PMC4747633

[B31] Messinger J. W. Trémeau F. Antonius D. Mendelsohn E. Prudent V. Stanford A. D. (2011). Avolition and expressive deficits capture negative symptom phenomenology: implications for DSM-5 and schizophrenia research. Clin. Psychol. Rev. 31, 161–168 10.1016/j.cpr.2010.09.002 20889248 PMC2997909

[B32] Miller J. Schwarz W. (2014). Brain signals do not demonstrate unconscious decision making: an interpretation based on graded conscious awareness. Conscious. Cogn. 24, 12–21 10.1016/j.concog.2013.12.004 24394375

[B33] Mogi K. (2013). Cognitive factors correlating with the metacognition of the phenomenal properties of experience. Sci. Rep. 3:3354 10.1038/srep03354 24284832 PMC3842081

[B34] Penfield W. (1975). The Mystery of the Mind: A Critical Study of Consciousness and the Human Brain. Princeton: Princeton University Press

[B35] Persinger M. A. (1983). Religious and mystical experiences as artifacts of temporal lobe function: a general hypothesis. Percept. Motor Skills 57, 1255–1262 10.2466/pms.1983.57.3f.1255 6664802

[B36] Pizzagalli D. Lehmann D. Gianotti L. Koenig T. Tanaka H. Wackermann J. (2000). Brain electric correlates of strong belief in paranormal phenomena: intracerebral EEG source and regional Omega complexity analyses. Psychiatry Res. 100, 139–154 10.1016/S0925-4927(00)00070-6 11120441

[B37] Rakos R. F. (2004). The belief in free will as a biological adaptation: thinking inside and outside the behavior analytic box. Eur. J. Behav. Analysis 5, 95–103

[B38] Rakos R. F. Laurence K. R. Skala S. Slane S. (2008). Belief in free will: measurement and conceptualization innovations. Behav, Soc. Issues 17, 20–39 10.5210/bsi.v17i1.1929

[B39] Reinders A. A. T. S. Nijenhuis E. R. Paans A. M. Korf J. Willemsen A. T. M. Den Boer J. A. (2003). One brain, two selves. Neuroimage 20, 2119–2125 10.1016/j.neuroimage.2003.08.021 14683715

[B40] Rice T. W. (2003). Believe it or not: religious and other paranormal beliefs in the United States. J. Sci. Study Relig. 42, 95–106 10.1111/1468-5906.00163

[B41] Romo R. Schultz W. (1987). Neuronal activity preceding self-initiated or externally timed arm movements in area 6 of monkey cortex. Exp. Brain Rese. 67, 656–662 10.1007/BF00247297 3653323

[B42] Schurger A. Sitt J. D. Dehaene S. (2012). An accumulator model for spontaneous neural activity prior to self-initiated movement. Proc. Natl. Acad. Sci. U.S.A. 109, E2904–E2913 10.1073/pnas.1210467109 22869750 PMC3479453

[B43] Smith K. (2011). Taking aim at free will. Nature 477, 23–25 10.1038/477023a 21886139

[B44] Strickland L. H. Lewicki R. J. Katz A. M. (1966). Temporal orientation and perceived control as determinants of risk-taking. J. Exp. Soc. Psychol. 2, 143–151 10.1016/0022-1031(66)90075-8

[B45] Taylor S. E. Brown J. D. (1988). Illusion and well-being: a social psychological perspective on mental health. Psychol. Bull. 103, 193–210 10.1037/0033-2909.103.2.193 3283814

[B46] Taylor S. E. Brown J. D. (1994). Positive illusions and well-being revisited: separating fact from fiction. Psychol. Bull. 116, 21–27 10.1037/0033-2909.116.1.21 8078971

[B47] Thompson S. C. (1999). Illusions of control: How we overestimate our personal influence. Curr. Dir. Psychol. Sci. 8, 187–190 10.1111/1467-8721.000446694056

[B48] Tobacyk J. J. (2004). A revised paranormal belief scale. Int. J. Transpers. Stud. 23, 94–98

[B49] Tobacyk J. Milford G. (1983). Belief in paranormal phenomena: Assessment instrument development and implications for personality functioning. J. Pers. Soc. Psychol. 44, 1029–1037 10.1037/0022-3514.44.5.1029

[B50] Van Inwagen P. (1975). The incompatibility of free will and determinism. Philos. Stud. 27, 185–199 10.1007/BF01624156

[B51] Viney W. Waldman D. A. Barchilon J. (1982). Attitudes toward punishment in relation to beliefs in free will and determinism. Hum. Relat. 35, 939–949 10.1177/001872678203501101

[B52] Wegner D. M. (2002). The Illusion of Conscious Will. Cambridge: MIT press

[B53] Wegner D. M. (2003). The mind's best trick: how we experience conscious will. Trends Cogn. Sci. 7, 65–69 10.1016/S1364-6613(03)00002-0 12584024

[B54] Wegner D. M. Fuller V. A. Sparrow B. (2003). Clever hands: uncontrolled intelligence in facilitated communication. J. Pers. Soc. Psychol. 85, 5–19 10.1037/0022-3514.85.1.5 12872881

[B55] Wegner D. M. Wheatley T. (1999). Apparent mental causation: Sources of the experience of will. Am. Psychol. 54, 480–492 10.1037/0003-066X.54.7.480 10424155

